# Unravelling spatial gene associations with SEAGAL: a Python package for spatial transcriptomics data analysis and visualization

**DOI:** 10.1093/bioinformatics/btad431

**Published:** 2023-07-12

**Authors:** Linhua Wang, Chaozhong Liu, Yang Gao, Xiang H -F Zhang, Zhandong Liu

**Affiliations:** Graduate School of Biomedical Sciences, Program in Quantitative and Computational Biosciences, Baylor College of Medicine, 1 Baylor Plaza, Houston, TX 77030, United States; Graduate School of Biomedical Sciences, Program in Quantitative and Computational Biosciences, Baylor College of Medicine, 1 Baylor Plaza, Houston, TX 77030, United States; Lester and Sue Smith Breast Center, Baylor College of Medicine, 1 Baylor Plaza, Houston, TX 77030, United States; Lester and Sue Smith Breast Center, Baylor College of Medicine, 1 Baylor Plaza, Houston, TX 77030, United States; Department of Molecular and Cellular Biology, Baylor College of Medicine, 1 Baylor Plaza, Houston, TX 77030, United States; McNair Medical Institute, Baylor College of Medicine, 1 Baylor Plaza, Houston, TX 77030, United States; Jan and Dan Duncan Neurological Research Institute at Texas Children’s Hospital, 1250 Moursund St, Houston, TX 77030, United States; Department of Pediatrics, Baylor College of Medicine, 1 Baylor Plaza, Houston, TX 77030, United States

## Abstract

**Summary:**

In the era where transcriptome profiling moves toward single-cell and spatial resolutions, the traditional co-expression analysis lacks the power to fully utilize such rich information to unravel spatial gene associations. Here, we present a Python package called Spatial Enrichment Analysis of Gene Associations using *L*-index (SEAGAL) to detect and visualize spatial gene correlations at both single-gene and gene-set levels. Our package takes spatial transcriptomics datasets with gene expression and the aligned spatial coordinates as input. It allows for analyzing and visualizing genes’ spatial correlations and cell types’ colocalization within the precise spatial context. The output could be visualized as volcano plots and heatmaps with a few lines of code, thus providing an easy-yet-comprehensive tool for mining spatial gene associations.

**Availability and implementation:**

The Python package SEAGAL can be installed using pip: https://pypi.org/project/seagal/. The source code and step-by-step tutorials are available at: https://github.com/linhuawang/SEAGAL.

## 1 Introduction

Spatial Transcriptomics (ST) is a molecular profiling technique that maps gene expression across a tissue sample, offering a comprehensive overview of gene expression patterns and unique biological insights (Marx 2021). The technique has applications in various fields, such as developmental biology (Choe *et al.* 2023), neuroscience (Close *et al.* 2021), and cancer research (Anderson *et al.* 2022).

To aid in data preprocessing and analysis, a variety of computational tools have been developed. Among these are SpatialDE (Svensson *et al.* 2018) and SquidPy (Palla *et al.* 2022), which identify Spatial Variable Genes (SVGs) through variance decomposition or spatial autocorrelation. These tools bring the concept of Highly Variable Genes (HVGs) from single-cell analysis to spatial transcriptomics. However, these SVG tools do not account for spatial associations such as colocalization and exclusion of paired features (genes or gene groups). Such spatial associations would add another dimension to traditional correlation-based co-expression analysis methods like the Weighted Correlation Network Analysis (Langfelder and Horvath 2008).

To fill the gap, we developed SEAGAL for Spatial Enrichment Analysis of Gene Associations using *L*-index, a bivariate spatial association measure proposed by (Lee 2001). Based on the *L*-index, SEAGAL allows ST data preprocessing, SVG detection, Spatially Associated Genes (SAG) identification, cell-type colocalization pattern recognition in local tissue niches, SAG-based gene module discovery, and so on.

SEAGAL is written in Python and compatible with well-established single-cell and spatial-omics analytical packages such as Scanpy (Wolf *et al.* 2018) and SquidPy (Palla *et al.* 2022). Moreover, integrated with well-supported Python visualization packages, including matplotlib and seaborn, SEAGAL allows visualizing each data analysis result in spatial heat maps, violin plots, and cluster maps. Its major functionalities could be carried out in a few lines of code by following the user manual and tutorials available at GitHub: https://github.com/linhuawang/SEAGAL.

## 2 Package description

SEAGAL takes inputs from either of the two types: (i) Raw 10X Visium data output from *SpaceRanger*, and (ii) a user-processed folder containing CSV-format files, including a raw count matrix, and metadata with *x* and *y* coordinates as columns. After importing the module *seagal*, users can load either type of input using the command load_raw().

- *Preprocess*
  As a quality control step, SEAGAL removes genes observed in <10 spots and spots with <150 UMI counts. To adjust for library size and normalize the variance, it transforms the raw counts into log-scaled count-per-million data types. Both steps are completed by calling the function process_st().
- *Cell-type colocalization*
group_adata_by_genes() followed by spatial_association() allow users to group gene markers to identify cell types' spatial associations, such as colocalization of two cell types. Users could either use default immune markers provided by SEAGAL (Supplementary Methods, Supplementary Fig. S1, Supplementary Table S1) or provide their cell-type marker dictionary as an optional parameter.
- *Gene–gene spatial association*
  To detect gene-gene spatial associations, users must first call spatial_pattern_genes() to detect SVGs. We recommend using <1000 top SVGs or SVGs with Moran’s *I* > 0.3 by specifying parameters *topK* or *I*. Then, spatial_association(grouped_only=False) will yield the spatial gene association in local and global *L*-values for the selected top SVG pairs (Supplementary Methods). Local *L* measures spatial association for each gene pair and each spot. It quantifies a spot’s spatial gene-gene association within its local neighborhood. A local *L* close to zero is considered as no spatial association, a positive value indicates positive correlation, and a negative value indicates negative correlation values. Global *L* is the mean value of all local *L*-index for a specific gene pair across all spots. It represents the general spatial association pattern between the two genes within the tissue. After calling this function, SEAGAL will output the global *L*-index for each gene-gene pair, the significance level through permutation test, and the corrected *P*-value using FDR correction by Benjamini–Hochberg (Benjamini and Hochberg 1995).
- *Gene module detection*
  Function genemodules() allows automatic gene module identification by finding the number of clusters that maximizes the silhouette score in iterative hierarchical clustering runs (Supplementary Methods).
- *Visualization*
  - General spatial associations could be visualized in a volcano plot and facilitate users to select their SAGs using volcano().
  - Spatial heat map of gene-gene association or cell-type colocalization for paired variables could be visualized by hotspot().
  - Summarized cell-type colocalization patterns could be visualized in a clustered heat map via clustermap().
  - Gene modules' expression patterns could be visualized through module_pattern(). And module_hotspot() plots module-module associations in spatial heat maps.

## 3 Example

In this section, we will use a 10X Visium dataset as an example to explore SEAGAL’s various functionalities. Other tutorials, including how to use CSV-format input or how to use user-defined marker gene lists for exploring cell–cell colocalization and exclusion are available at: https://github.com/linhuawang/SEAGAL.

The Human Breast Cancer block 1 sample (HBC1) originated from 10X Genomics and could also be downloaded from our example datasets: https://github.com/linhuawang/SEAGAL.

**Figure 1.**
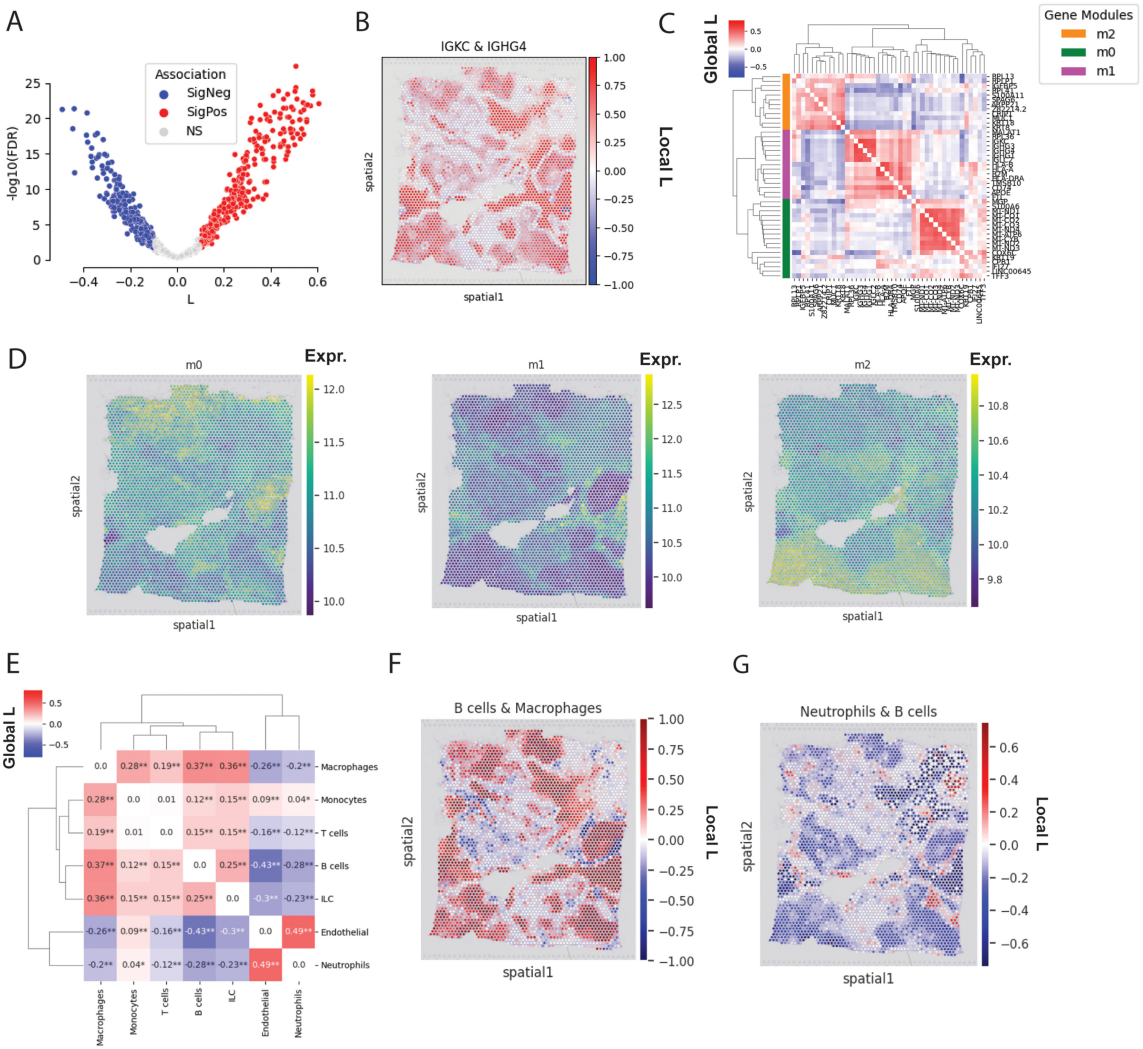
Applying SEAGAL on a breast cancer tumor. (A) Volcano plot of Spatially Associated Gene (SAG) pairs; each dot is a pair of spatially variable genes; *x* axis shows the global spatial association in *L*-index and *y* axis shows the –log10 of *P*-value corrected by FDR using Benjamini–Hochberg’s approach in permutation test. (B) Spatial heat map showing niches where a pair of SAGs are colocalized based on the local *L*-index. (C) Clustered heatmap with gene modules annotated in each row. (D) Detected modules’ expression patterns in spatial heat maps. (E) Clustered heatmap for immune cell types’ spatial associations. Spatial heat map showing the spatial niches where B cells and Macrophages are spatially colocalized (F) and B cells and Neutrophils are spatially excluded (G) based on the local *L*-index

The core functionality of SEAGAL is to detect spatially associated gene pairs, as illustrated in Fig. 1A. This volcano plot displays the spatial associations identified by SEAGAL using highly spatially variable genes with Moran's I ≥ 0.4. The gene pairs that show significant association are consistent with other methods used to quantify spatial gene co-expression values for spatial transcriptomics (Supplementary Fig. S1). Notably, the gene pair with the highest positive global *L*-value (IGKC & IGHG) exhibits similar spatial expression patterns, whereas the gene pair with the largest negative *L*-value (COX6C & RPL13) demonstrates exclusive spatial expression patterns (Supplementary Fig. S2). Subsequently, SEAGAL was employed to calculate local *L*-values, representing the correlation of gene pairs within the spatial neighborhood of each spot. The heatmap (Fig. 1B, Supplementary Figs S2–S4) highlights the spatially correlated niches, providing crucial insights into the intricate spatial organization and gene associations within the tissue, which are instrumental in deciphering complex biological processes and understanding the underlying mechanisms at play.

Inspired by the Weighted Gene Co-expression Network Analysis (WGCNA) analysis, SEAGAL enables the identification of spatially co-expressed gene modules that exhibit co-expression patterns specific to certain spatial locations. These gene modules indicate the dynamics of gene groups or pathways within precise spatial contexts. In the example presented, SEAGAL identified three Spatially Associated Gene (SAG) modules (Fig. 1C), each displaying distinct expression spatial patterns (Fig. 1D). The module assignments agree with results from Giotto (Dries *et al.* 2021), yielding an adjusted Rand index of 0.74 (Supplementary Fig. S5).

Furthermore, gene set enrichment analysis revealed that the three identified modules are over-represented by different functions in terms of gene ontology or cancer cell lines (Supplementary Fig. S6). Specifically, module m0 is enriched with the Breast Cancer cell line BT483, as defined by the Cancer Cell Line Encyclopedia (Ghandi *et al.* 2019). Module m1 is associated with signaling pathways related to B and T cells, while module m2 is predominantly linked to gene ontologies related to cell migration and adhesion. This divergence in the molecular functional associations of the modules reflects the dynamic pathways and cell communications within the underlying tissue.

To investigate how immune cells communicate within the breast cancer tissue, we utilized SEAGAL to assess the colocalization patterns of immune cells. We grouped the expression of marker genes together and represented the paired cell–cell colocalization using global *L*-values (Fig. 1E). To validate our findings, we compared them to the results obtained from cell-type deconvolution using RCTD, demonstrating a strong agreement (Pearson's correlation = 0.69, *P* = 0.0005) (Supplementary Fig. S7).

Our analysis revealed intriguing associations in the spatial distribution of immune cells. B cells exhibited a spatial colocalization with macrophages (global *L* = 0.37) while showing a negative association with neutrophils (global *L* = –0.28). Local *L*-values displayed spatial patterns indicating positive correlations between B cells and macrophages and negative correlations between B cells and neutrophils across most of the tissue area. However, there were areas within the tissue where only one specific association existed, or none of the associations were present (Fig. 1F and G).

These findings highlight the diversity and dynamics of immune cell–cell interactions throughout the tumor tissue, underscoring the intra-tumor heterogeneity of immune responses. Importantly, the spatial distribution of immune cells in the tumor microenvironment significantly influences their functional interactions and their ability to target cancer cells effectively (Bindea *et al.* 2013). SEAGAL’s cell-type colocalization feature facilitates the exploration of colocalization or exclusion of different immune cells within precise spatial contexts. This capability can potentially enhance the development of more effective therapeutic strategies.

## Supplementary Material

btad431_Supplementary_Data

## Data Availability

The data underlying this study are available in GitHub at https://github.com/linhuawang/SEAGAL/tree/main/test/data/VisiumData/Human_Breast_Cancer_BAS1.
