# Supplementary material for: Unravelling spatial gene associations with SEAGAL: a Python package for spatial transcriptomics data analysis and visualization
Source: Bioinformatics. 2023 Jul 12;39(7):btad431. doi: 10.1093/bioinformatics/btad431 (PMC10363022; doi:10.1093/bioinformatics/btad431)
Supplement: btad431_Supplementary_Data [file btad431_supplementary_data.pdf]

Supplementary Methods

Extracting Spatially Variable Gene (SVG)

To quantify the spatial variation of each gene, we calculated Moran's *I* for gene **x** of interest defined as:

$$I = \frac{N}{S}\frac{\sum_{i=1}^{N}\sum_{j=1}^{N} w_{ij}(x_i - \bar{x})(x_j - \bar{x})}{\sum_{i=1}^{N}(x_i - \bar{x})^2}$$

where **N** is the number of spots within the tissue, w_{ij} represents the spatial weight between spot pair <*i, j*>, **S** is the number of such pairs of spots. Instead of implementing Moran's I from scratch, SEAGAL incorporated the function *squidpy.gr.spatial_autocorr()* written in SquidPy [1].

After calculating the spatial variation of each gene denoted by Moran's *I*, SEAGAL ranks genes by their values of *I*. Users either select a cutoff of *I* or the number of top highly spatially variable genes for further Spatially Associated Gene (SAG) analysis.

Calculation of local L index and global L index

We introduced the spatial correlation measure in geographical studies [2] and fitted it to the spatial transcriptomics studies. This correlation, named the *L* index, utilizes the spatial lag concept to quantify the local correlation value for all spots within the tissue. This local value indicates a spot's gene co-expression within its local neighborhood. Local L index of cell i between gene **x** and gene **y** is defined as:

$$L_{x,y}^{(i)} = \frac{n \cdot (\tilde{x}_i - \bar{x})(\tilde{y}_i - \bar{y})}{\sqrt{\sum_i (x_i - \bar{x})^2}\sqrt{\sum_i (y_i - \bar{y})^2}}$$

where $\bar{x}$ and $\bar{y}$ are the numeric mean values of **x** and **y**, $\tilde{\mathbf{x}}$ and $\tilde{\mathbf{y}}$ are the spatial lag values composed of weighted averages of dot neighbors. Spatial lag of x_i is defined as:

$$\tilde{x}_i = \sum_j w_{ij} \cdot x_j$$

where j is connected dots with i in the space, and w_{ij} is their connectivity weight. Here, we take the spatial connection as the connectivity matrix $\mathbf{W}$, which was obtained from the function *spatial_neighbors( )* from the *Squidpy* Python package. For Visium data, it takes the first hexagonal ring around the spot as the neighbors and assigns equal weights to each neighbor. Before calculating any L index, we normalize the W matrix by dividing it by the row sums. A global *L* index is calculated as the average local *L* index for all the spots within the tissue to represent the average spatial correlation between gene **x** and gene **y.**

Permutation-based p-value calculation for L index

The significance tests of both global and local L index are from the permutation approach. The paired vectors were shuffled together n times to get a reference distribution of L index. Then the

z-score of L index under the reference distribution is calculated and taken as the simulated p-value (see pseudo-code below). Moreover, SEAGAL p-values were corrected for multiple tests by False Discovery Rate (FDR) using Benjamini-Hochberg approach. The FDR correction is implemented by calling the function *statsmodels.stats.multitest.fdrcorrection().* To account for FDR correction without the assumption on independency of test statistics, we provided a parameter "*indep*" in the function for the users to indicate independency of the tests. If "*indep*" is set to be True, BH correction will be used, otherwise, FDR correction by Benjamini-Yekutieli will be used.

Algorithm Significance test for global L index

for $x \in \mathbb{R}^n, y \in \mathbb{R}^n$

L_{ref} = [ ] (Reference distribution of $L_{x,y}$)

repeat n times

  x', y' = **shuffle** 10% x, y together

  append $L_{x'y'}$ **to** L_{ref}

return $p = \frac{L_{x,y} - \bar{L}_{ref}}{std(L_{ref})}$

Due to the time-consuming process of permutation test, we recommend users use a Moran's I threshold of greater than 0.3 or select top 1000 SVGs for SAG analysis. They could be specified as parameters *I* or *topK* of function *seagal.spatial_pattern_genes().*

Gene module detection

To group genes based on spatial correlation for users' further investigation, we borrowed the idea from Weighted Gene Co-expression Network Analysis (WGCNA) to detect gene modules. After having a Spatial Association Gene matrix, denoted as $\mathbf{X} \in \mathbf{R}^{k \times k}$, where k is the number of top SVGs ranked by Moran's *I*. The algorithm below illustrates how the number of gene modules were optimized.

Algorithm Gene module assignment

s_{opt} = -1 #initiate Silhouette score
$y_{opt} = 1, \ldots, 1$ #assign initial labels
for i = 1 to n
  $y_i = HierarchicalCluster(\mathbf{X}, i)$
  $s_i = SilhouetteScore(\mathbf{X}, y_i)$
  if $s_i > s_{opt}$:
    $y_{opt} = y_i$
    $s_{opt} = s_i$
return y_{opt}

In the gene module assignment algorithm, we used the Python function *AgglomerativeClustering()* from the *sklearn.cluster* module to perform hierarchical clustering. We used the function *silhouette_score()* from the *sklearn.metrics* module to estimate the clustering results' accuracy in Silhouette score.

Immune single-cell RNA-seq data collection and preprocessing
Blood and tumor samples were harvested from PyMT-M tumor-bearing mice. Blood samples (n=3) are collected retro-orbitally using caliper tubes and processed with red blood cell lysis buffer (Tonbo Biosciences) before library preparation. A tumor sample are dissociated with Tumor Dissociation Kit following the manufacturer's instructions (Miltenyi Biotec). After isolation and filtering through a 70µm filter, CD45+DAPI- cells were sorted using FACSAria cell sorter (BD Biosciences) at the Cytometry and Cell Sorting Core. The single-cell libraries were prepared using Chromium Controller (10X Genomics) at the Single Cell Genomics Core and sequenced using NovaSeq 6000 at the Genomics and RNA Profiling Core of Baylor College of Medicine. The FASTQ files were processed using Cell Ranger pipelines (10X Genomics) to generate feature-barcode matrices.

Integrating immune single-cell RNA-seq data from the blood and tumor of PyMT-M mouse
We followed the Seurat [3] tutorial on single-cell RNA integration from https://satijalab.org/seurat/articles/integration_introduction.html. Specifically, both datasets were library-size normalized and log scaled. Then, variable genes from both datasets were extracted, and overlapped variable genes were used as anchors to integrate the two datasets to generate a combined immune single-cell RNA-seq dataset.

Immune cell type annotation using SingleR
After having the integrated immune single-cell RNA-sequencing data, we performed the standard pipeline for clustering including scaling the expression data, performing dimension

reduction using PCA and UMAP, and finding clusters by a shared nearest neighbor (SNN) modularity optimization (https://satijalab.org/seurat/articles/integration_introduction.html). Then, we used the R package *SingleR* [4] to assign cell type labels to each of the identified cluster using the *ImmGen* reference data from the Immunological Genome Project [5]. Supp. Figure 9 shows the UMAP with the data source (blood/tumor) and the assigned cell type labels from *SingleR*.

Extracting immune signatures for immune colocalization analysis

After annotating the cell types for the integrated single cell RNA-seq data, we extracted the top-20 significantly enriched genes as cell type-specific markers. Specifically, we used the *FindAllMarkers()* function from *Seurat* [3] to calculate the fold change and significance level for each cell type as compared with all other cell types. Then, we filtered genes with fold change less than 20% or adjusted p-value greater than 0.05. Finally, for each cell type, we extracted 20 genes that have the largest average fold changes as cell type markers. The resulted marker gene list is shown in Supp. Table 1.

Immune colocalization and exclusion

It is known that single-cell RNA-seq is highly sparse due to a shallow sequencing-depth that causes dropout and excessive zero-values [6]. By grouping top-k (k=20 by default) cell-type marker genes, SEAGAL allows to amplify cell type marker signals that would otherwise be obscured using one or few signature genes. Motivated by this, SEAGAL allows grouping the marker genes either from the default immune marker list (Supp. Table 1) or from the user input. Specifically, the raw gene counts for top-k markers of each cell type group were aggregated together before library size normalization and log scaled. Then, SEAGAL performs bivariate spatial correlation analysis for each pair of such groups. As a result, users could either visualize the local spatial correlation denoted local L index or summarize the overall spatial correlation within the tissue using the global L index, which is essentially the average local L values across all spots.

Supplementary Figures

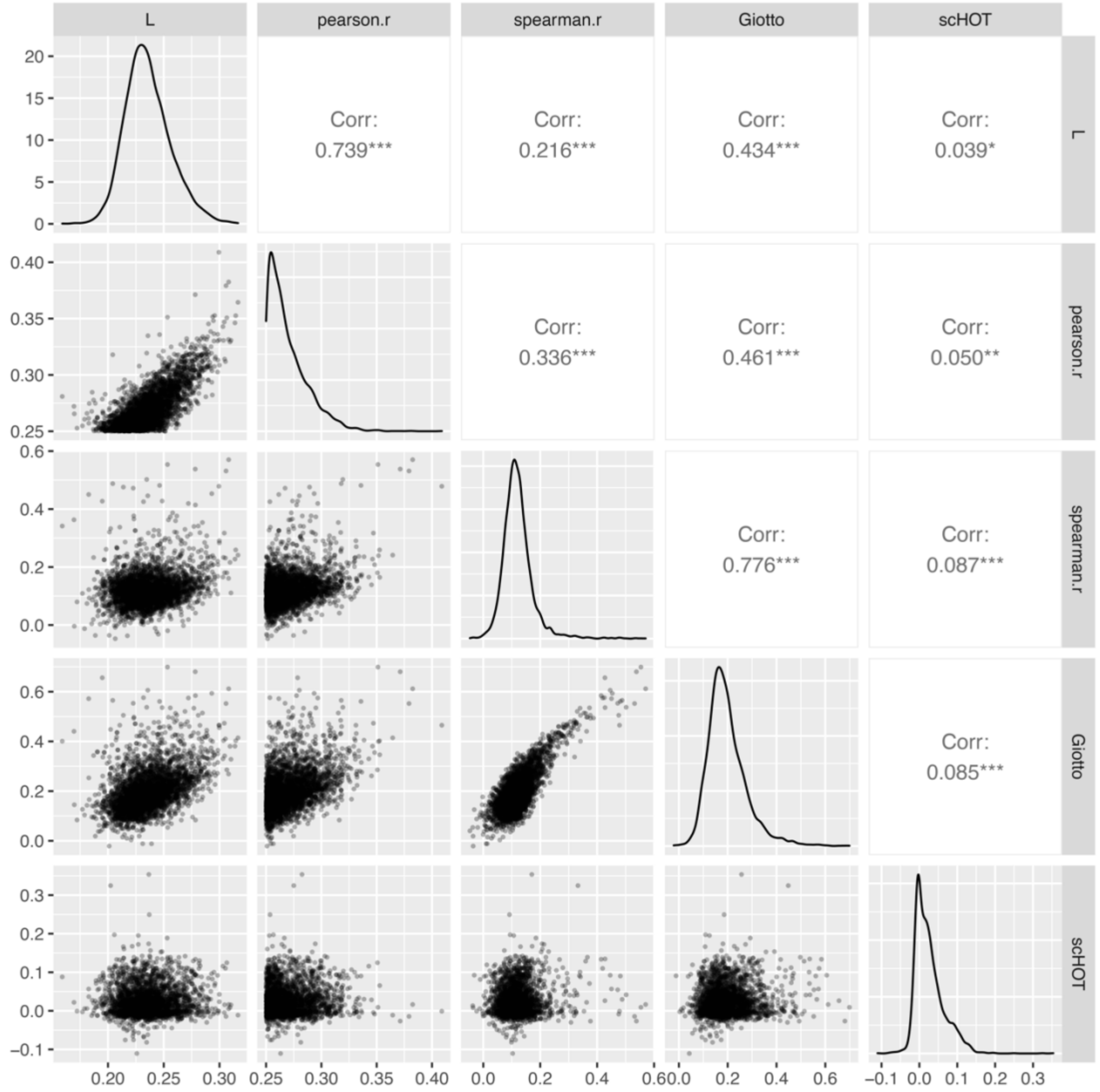

Supplementary Figure 1 Benchmarking SEAGAL's global L against other methods in quantifying spatial coexpression of gene pairs at the tissue level. Methods compared include Pearson's and Spearman's correlations, Giotto [7], and scHOT [8]. In total, 3759 gene pairs with at least 0.25 Pearson's correlation were randomly selected for comparison.

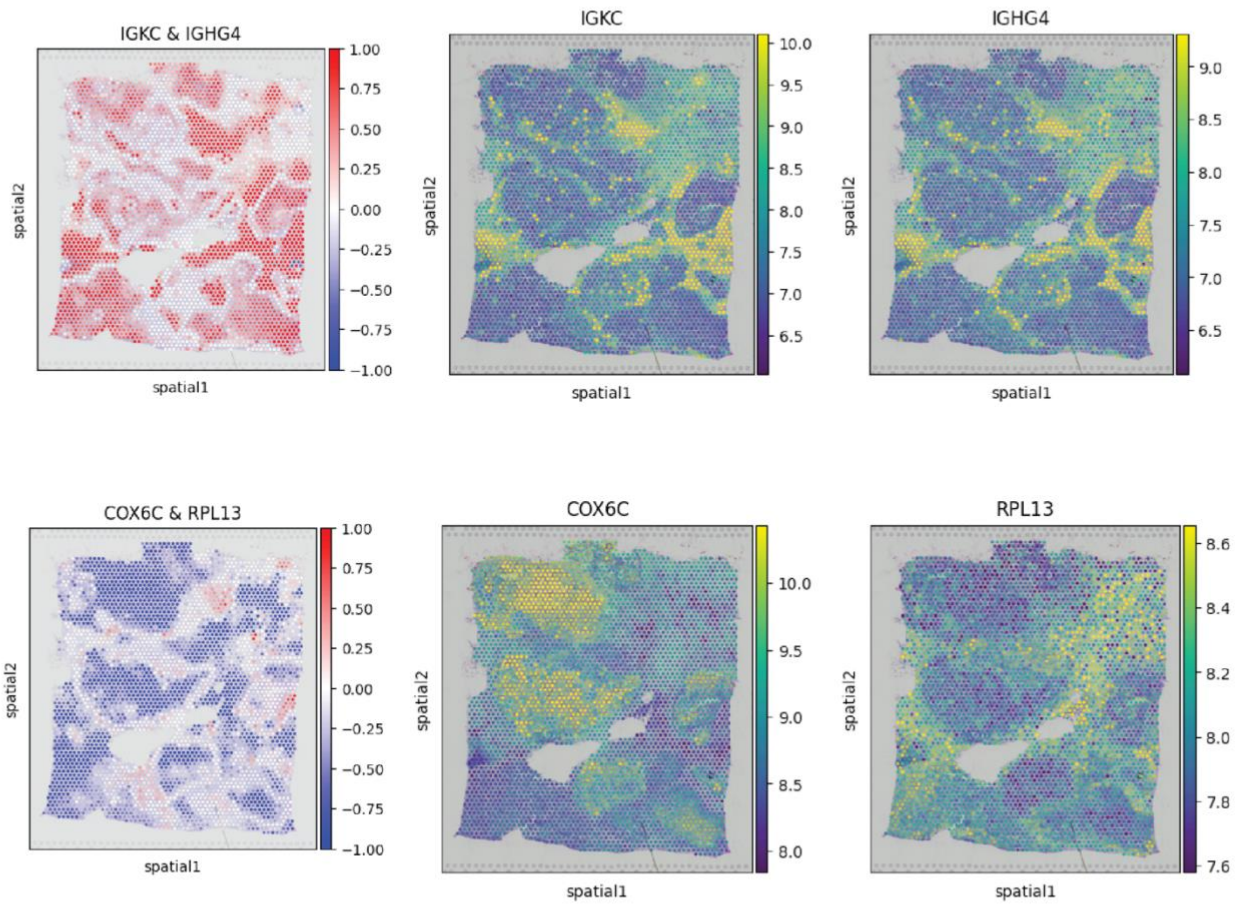

Supplementary Figure 2 Gene pairs with the largest positive L and negative L values. Top: IGKC & IGHG4 (global L = 0.61), bottom: COX6C & RPL13 (global L = -0.49). Column 1: spatial heat map of local L values. Columns 2-3: expression heat maps of each gene.

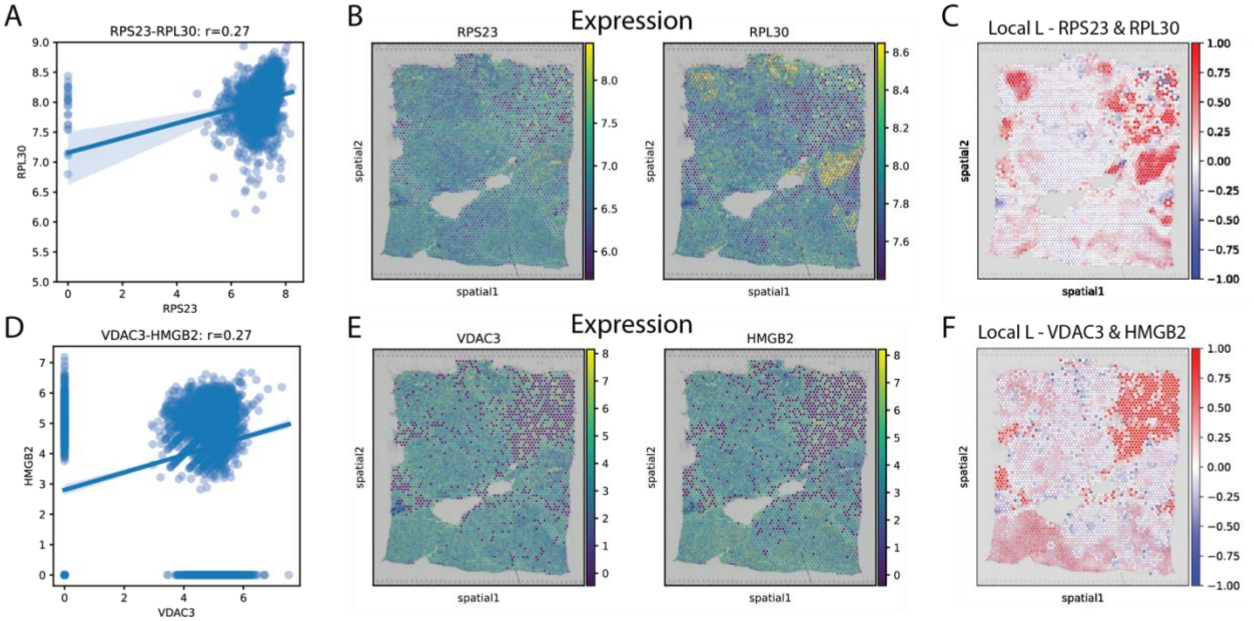

Supplementary Figure 3 SEAGAL's L prioritizes the gene pair with better spatial patterns among gene pairs with the same Pearson's correlation scores. A Correlation of RPS23-RPL30 in scatter plots with Pearson's correlation of 0.27. **B** Spatial expression heat maps of genes RPS23 and RPL30. **C** Local L heat map of the gene pair RPS23 and RPL30 (global L. = 0.17). **D** Correlation of VDAC3-HMGB2 in scatter plots with Pearson's correlation of 0.27. **E** Spatial expression heat maps of genes VDAC3 and HMGB2. **F** Local L heat map of the gene pair VDAC3 and HMGB2 (global L. = 0.26).

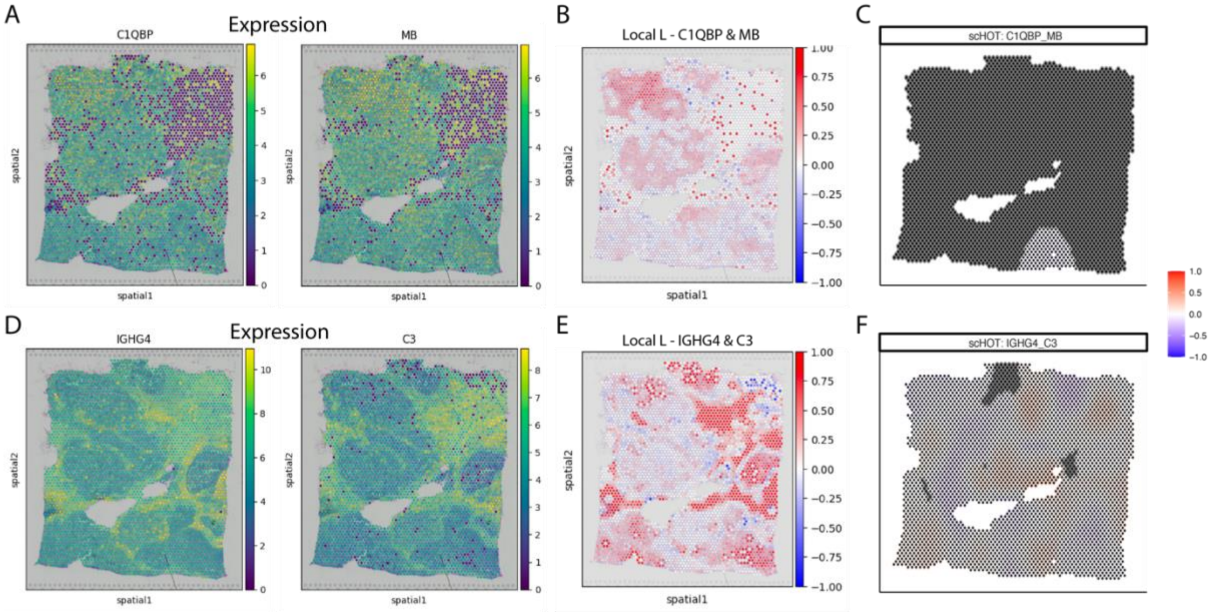

Supplementary Figure 4 SEAGAL's L recovered spatially correlated gene pairs missed by scHOT. A Spatial expression heat maps of genes C1BP and MP. **B** Local L heat map of the gene pair C1BP and MP (global L=0.32). **C** scHOT's local correlation heat map of the gene pair C1BP and MP (scHOT spatial correlation = -0.011, black dot indicates "NA" values from the scHOT's output). **D** Spatial expression heat maps of genes IGHG4 and C3. **E** Local L heat map of the gene pair IGHG4 and C3 (global L = 0.31). **F** scHOT's local correlation heat map of the gene pair IGHG4 and C3 (scHOT spatial correlation = -0.017).

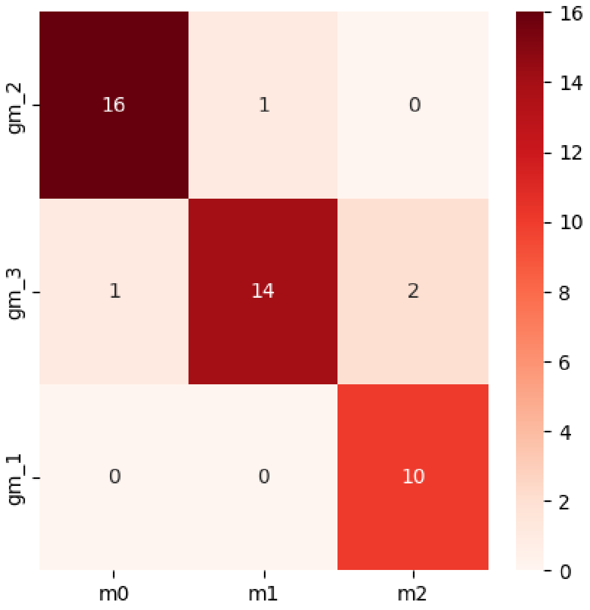

Supplementary Figure 5 Confusion matrix of module assignment agreement between Giotto and SEAGAL. Heat map of the intersection of module assignments for the highly spatially variable genes (moran's I >= 0.4, n=44). Rows are the module assignments from Giotto and columns are module assignment using SEAGAL.

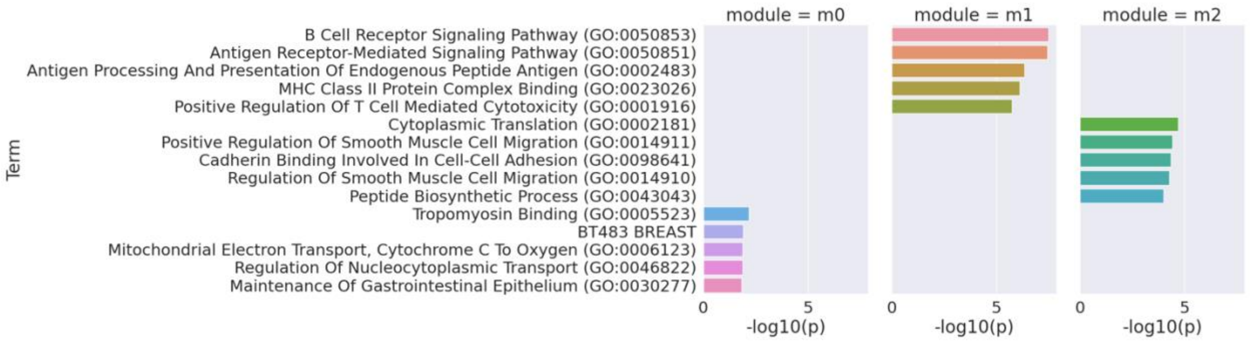

Supplementary Figure 6 Top-5 enriched pathways for each module using Gene Ontology and Cancer Cell Line Encyclopedia.

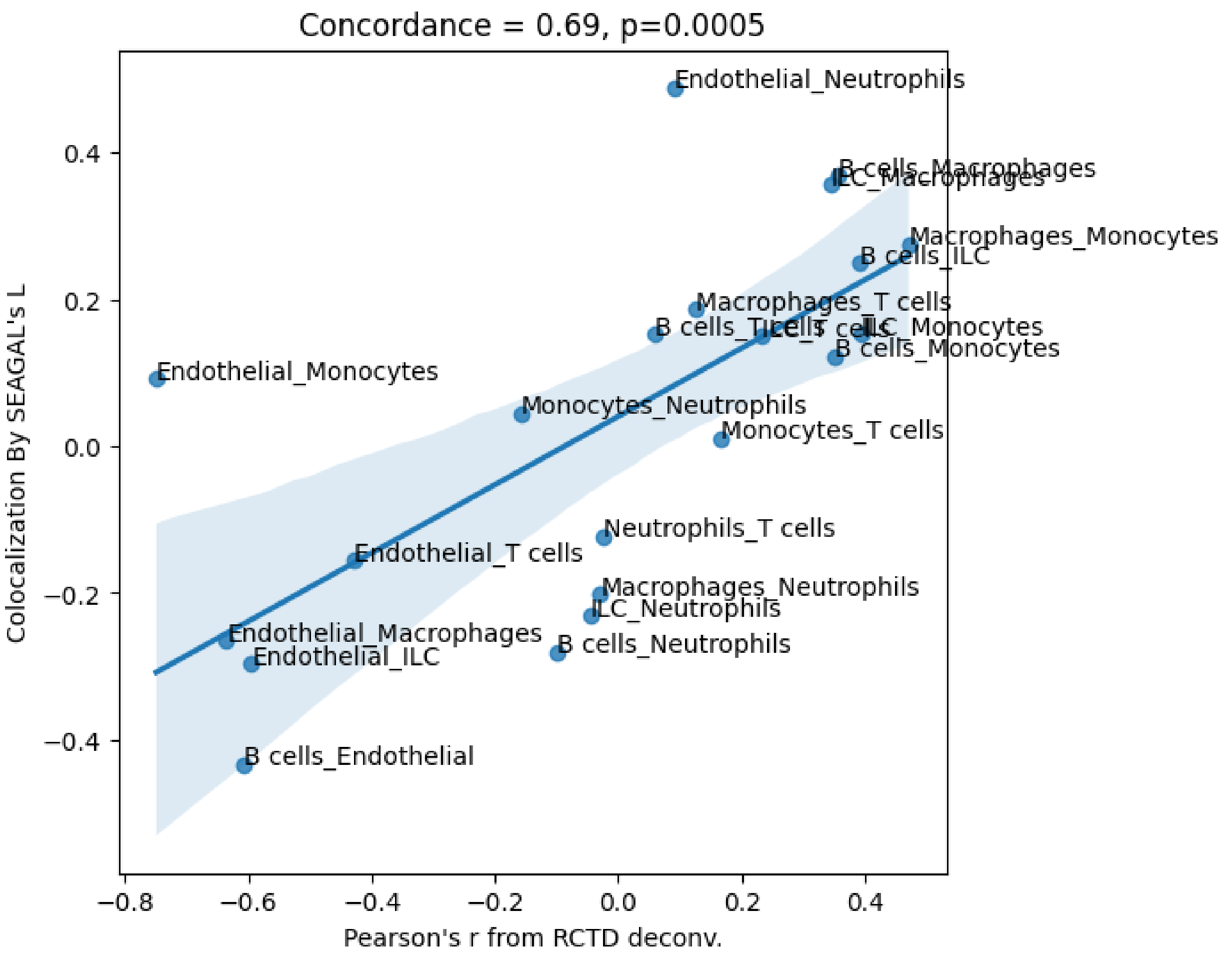

Supplementary Figure 7 Comparison between SEAGAL's cell type colocalization result and Pearson's correlation between pair-wise cell types' proportions from RCTD, a cell-type deconvolution approach for spatial transcriptomics. The concordance score is calculated by Pearson's correlation of results from both measures.

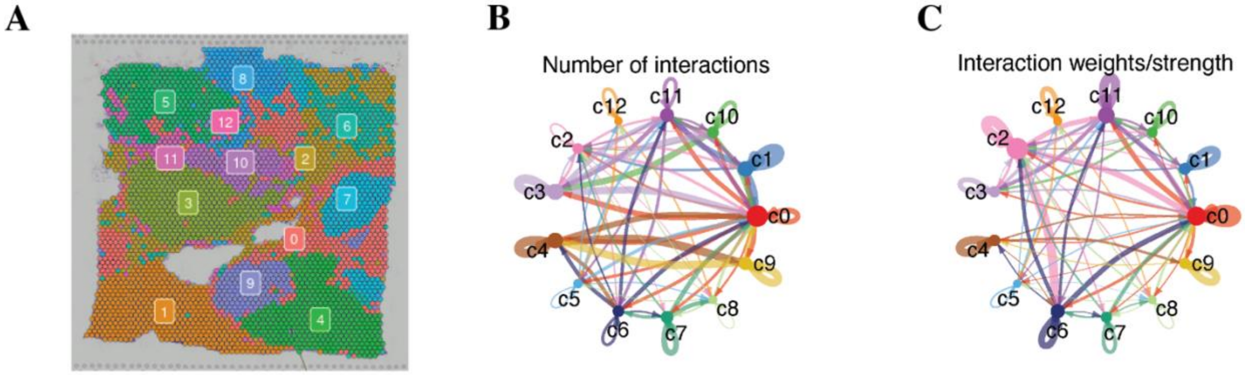

Supplementary Figure 8 Applying CellChat on the Breast Cancer tumor. A Clusters identified using Seurat. **B** Spot-spot interaction counts among clusters using CellChat. C Interaction weights/strength among clustered spots by CellChat.

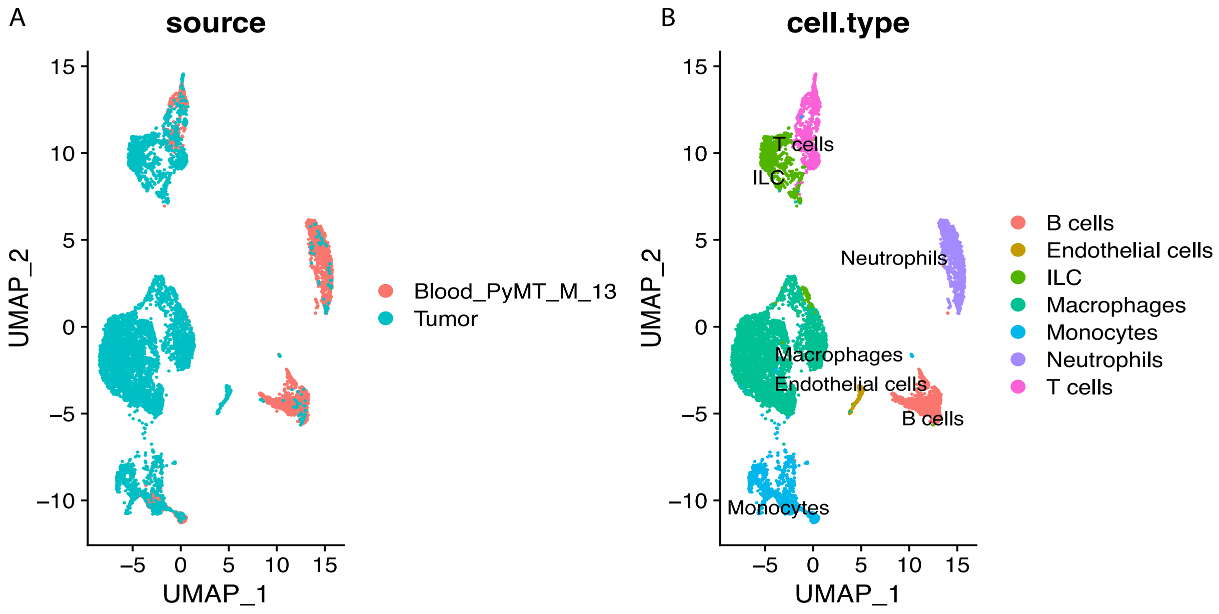

Supplementary Figure 9 UMAP of combined single-cell RNA-sequencing data colored by data source (A) or SingleR-assigned cell type labels (B).

Supplementary Table 1 Immune signature genes from single-cell RNA-seq data

B cells	IGKC	4.863283895	0	0
B cells	EBF1	4.853267878	0	0
B cells	BANK1	4.622753198	0	0
B cells	CD79A	4.570748748	0	0
B cells	IGHD	3.961262136	0	0
B cells	BACH2	3.644474971	0	0
B cells	IGHM	3.62593866	0	0
B cells	FCHSD2	3.609434401	0	0
B cells	LY6D	3.54306969	0	0
B cells	PAX5	3.488057181	0	0
B cells	RALGPS2	3.459272173	0	0
B cells	IGLC2	3.426724375	0	0
B cells	AFF3	3.363231126	0	0
B cells	MAN1A	3.325816329	0	0
B cells	BTLA	3.19322992	0	0
B cells	MEF2C	3.192447124	0	0
B cells	CD79B	3.185290189	0	0
B cells	MS4A1	3.184677546	0	0
B cells	FOXP1	3.159180956	0	0
B cells	CD55	3.08304872	0	0
Endothelial	WFDC18	7.516936759	9.64E-122	1.70E-117
Endothelial	CSN3	5.033715164	0	0
Endothelial	LCN2	4.946266553	9.11E-143	1.60E-138
Endothelial	PHLDA1	4.73546053	1.54E-264	2.70E-260
Endothelial	KRT18	4.130755865	0	0
Endothelial	MAP1LC3A	3.854317431	2.40E-96	4.22E-92
Endothelial	HRAS	3.655577476	2.46E-108	4.33E-104
Endothelial	AQP5	3.653494023	0	0
Endothelial	KRT8	3.60210388	0	0
Endothelial	CRIP2	3.163123894	0	0
Endothelial	CDKN1A	3.108054212	3.72E-67	6.55E-63
Endothelial	DBI	3.031617832	2.65E-45	4.66E-41
Endothelial	PGP	2.874093091	3.75E-62	6.59E-58
Endothelial	RPS27L	2.871893252	8.99E-62	1.58E-57
Endothelial	1110008P14RIK	2.732528641	4.13E-64	7.27E-60
Endothelial	SPINT2	2.715140378	6.16E-85	1.08E-80
Endothelial	MRPS6	2.704714303	6.49E-95	1.14E-90

Endothelial	DSTN	2.69910403	2.87E-47	5.06E-43
Endothelial	HMGN1	2.489873464	4.09E-45	7.19E-41
Endothelial	XBP1	2.396149574	9.07E-15	1.60E-10
ILC	CD7	4.735542968	0	0
ILC	GZMB	4.497932526	0	0
ILC	XCL1	3.836119906	0	0
ILC	IL2RB	3.236869249	0	0
ILC	NKG7	3.109126917	0	0
ILC	CTSW	2.879358468	0	0
ILC	CCL5	2.636434009	0	0
ILC	AW112010	2.412621725	0	0
ILC	KLRD1	2.337469899	0	0
ILC	TMSB10	2.298579567	0	0
ILC	KLRB1C	2.285921203	0	0
ILC	CAR2	2.063262973	0	0
ILC	KLRK1	2.042333188	0	0
ILC	KLRE1	2.036805993	0	0
ILC	CST7	2.032869701	0	0
ILC	NCR1	2.021255165	0	0
ILC	LCK	1.843598252	0	0
ILC	SERPINA3G	1.83935391	0	0
ILC	SH2D2A	1.785709721	0	0
ILC	KLRB1A	1.70520489	0	0
Macrophages	APOE	3.696588288	0	0
Macrophages	C1QA	3.579553924	0	0
Macrophages	C1QC	3.566715729	0	0
Macrophages	C1QB	3.528304657	0	0
Macrophages	MS4A7	3.49533347	0	0
Macrophages	CD81	3.444080754	0	0
Macrophages	CCL4	3.09772619	0	0
Macrophages	RGS1	2.962227119	0	0
Macrophages	CD72	2.91765483	0	0
Macrophages	CTSS	2.886982712	0	0
Macrophages	HEXB	2.846952019	0	0
Macrophages	CTSB	2.790360165	0	0
Macrophages	ACP5	2.757745342	0	0
Macrophages	CD63	2.706079998	0	0
Macrophages	LGMN	2.635374329	0	0
Macrophages	LY86	2.565704591	0	0

Macrophages	AIF1	2.562323509	0	0
Macrophages	GRN	2.551964928	0	0
Macrophages	PLD4	2.551367026	0	0
Macrophages	CXCL16	2.52469736	0	0
Monocytes	CRIP1	3.202530851	0	0
Monocytes	PLAC8	3.09487916	0	0
Monocytes	CCL6	2.894695562	2.93E-282	5.16E-278
Monocytes	S100A4	2.518317959	0	0
Monocytes	CCL9	2.440780729	0	0
Monocytes	F13A1	2.423940816	0	0
Monocytes	AHNAK	2.309121068	0	0
Monocytes	CCR2	2.204451114	0	0
Monocytes	IFITM3	2.183880461	0	0
Monocytes	VIM	2.096796228	1.86E-300	3.27E-296
Monocytes	ALOX5AP	1.938398879	0	0
Monocytes	IFITM6	1.886593664	0	0
Monocytes	PLTP	1.827150298	5.35E-88	9.42E-84
Monocytes	LYZ2	1.817068804	3.96E-235	6.97E-231
Monocytes	TAGLN2	1.659646112	9.27E-223	1.63E-218
Monocytes	IFI27L2A	1.654564384	1.39E-197	2.44E-193
Monocytes	GPX1	1.651578965	0	0
Monocytes	PID1	1.528488516	8.24E-260	1.45E-255
Monocytes	ANXA2	1.480303354	9.10E-277	1.60E-272
Monocytes	EMP3	1.476752441	5.93E-230	1.04E-225
Neutrophils	S100A8	8.111018837	0	0
Neutrophils	S100A9	8.025284102	0	0
Neutrophils	RETNLG	5.386795167	0	0
Neutrophils	CSF3R	4.91738625	0	0
Neutrophils	IFITM1	4.65612864	0	0
Neutrophils	SLPI	4.627605827	0	0
Neutrophils	HDC	4.389116225	0	0
Neutrophils	MMP9	4.059459951	0	0
Neutrophils	CXCR2	4.014865947	0	0
Neutrophils	PBX1	3.770408082	0	0
Neutrophils	GSR	3.702644054	0	0
Neutrophils	MXD1	3.637516414	0	0
Neutrophils	GDA	3.582710521	0	0
Neutrophils	SORL1	3.563383529	0	0
Neutrophils	CLEC4D	3.517811345	0	0

Neutrophils	WFDC21	3.502059557	0	0
Neutrophils	S100A11	3.407301716	0	0
Neutrophils	IL1R2	3.391184164	0	0
Neutrophils	CD44	3.378302268	0	0
Neutrophils	PGLYRP1	3.306221682	0	0
T cells	GM2682	3.902952905	0	0
T cells	LEF1	3.368509766	0	0
T cells	SKAP1	3.258969953	0	0
T cells	CD3E	3.021577364	0	0
T cells	MS4A4B	2.938070044	0	0
T cells	ITK	2.89086625	0	0
T cells	CD3G	2.834731067	0	0
T cells	CD3D	2.819694445	0	0
T cells	INPP4B	2.765241015	4.12E-247	7.25E-243
T cells	LAT	2.719651983	0	0
T cells	SATB1	2.670865674	1.85E-260	3.26E-256
T cells	PRKCQ	2.582773026	0	0
T cells	THY1	2.531753449	0	0
T cells	CD247	2.482211215	0	0
T cells	TRBC2	2.387395924	0	0
T cells	BCL11B	2.383388204	0	0
T cells	NKG7	2.333211168	1.16E-202	2.04E-198
T cells	GRAP2	2.292002958	2.32E-197	4.08E-193
T cells	TCF7	2.289982075	0	0
T cells	BCL2	2.242102505	1.58E-252	2.78E-248

References

1. Palla, G., Spitzer, H., Klein, M., Fischer, D., Schaar, A. C., Kuemmerle, L. B., Rybakov, S., Ibarra, I. L., Holmberg, O., Virshup, I., Lotfollahi, M., Richter, S., & Theis, F. J. (2022). Squidpy: A scalable framework for spatial omics analysis. *Nature Methods*, *19*(2), 171-178. https://doi.org/10.1038/s41592-021-01358-2
2. Lee,S.-I. (2001) Developing a bivariate spatial association measure: An integration of Pearson's r and Moran's I. J Geogr Syst, 3, 369–385.
3. Hao, Y., Hao, S., Andersen-Nissen, E., Zheng, S., Butler, A., Lee, M. J., Wilk, A. J., Darby, C., Zager, M., Hoffman, P., Stoeckius, M., Papalexi, E., Mimitou, E. P., Jain, J., Srivastava, A., Stuart, T., Fleming, L. M., Yeung, B., Rogers, A. J., . . . Satija, R. (2021). Integrated analysis of multimodal single-cell data. *Cell*, *184*(13), 3573-3587. https://doi.org/10.1016/j.cell.2021.04.048
4. Aran, D., Looney, A. P., Liu, L., Wu, E., Fong, V., Hsu, A., Chak, S., Naikawadi, R. P., Wolters, P. J., Abate, A. R., Butte, A. J., & Bhattacharya, M. (2019). Reference-based analysis of lung single-cell sequencing reveals a transitional profibrotic macrophage. *Nature Immunology*, *20*(2), 163-172. https://doi.org/10.1038/s41590-018-0276-y
5. Gainullina, A., Mogilenko, D. A., Huang, L. H., Todorov, H., Narang, V., Kim, K. W., Yng, L. S., Kent, A., Jia, B., Seddu, K., Krchma, K., Wu, J., Crozat, K., Tomasello, E., Dress, R., See, P., Scott, C., Gibbings, S., Bajpai, G., Desai, J. V., … ImmGen Consortium (2023). Network analysis of large-scale ImmGen and Tabula Muris datasets highlights metabolic diversity of tissue mononuclear phagocytes. *Cell reports*, *42*(2), 112046. Advance online publication. https://doi.org/10.1016/j.celrep.2023.112046https://doi.org/10.1016/j.celrep.2023.112046
6. Stegle, O., Teichmann, S. A., & Marioni, J. C. (2015). Computational and analytical challenges in single-cell transcriptomics. *Nature Reviews Genetics*, *16*(3), 133-145. https://doi.org/10.1038/nrg3833
7. Dries, R., Zhu, Q., Dong, R. *et al.* Giotto: a toolbox for integrative analysis and visualization of spatial expression data. *Genome Biol* **22**, 78 (2021). https://doi.org/10.1186/s13059-021-02286-2
8. Ghazanfar, S., Lin, Y., Su, X. *et al.* Investigating higher-order interactions in single-cell data with scHOT. *Nat Methods* **17**, 799–806 (2020). https://doi.org/10.1038/s41592-020-0885-x
